# Prevalence, epidemiology and molecular studies of *Tomato chlorosis virus* (ToCV) in South Africa

**DOI:** 10.1371/journal.pone.0220298

**Published:** 2019-07-24

**Authors:** Vaneson Moodley, Augustine Gubba, Paramu L. Mafongoya

**Affiliations:** 1 Department of Plant Pathology, School of Agricultural, Earth and Environmental Sciences, University of KwaZulu-Natal, Scottsville, Pietermaritzburg, South Africa; 2 Department of Rural Agronomy and Development, School of Agricultural, Earth and Environmental Sciences, University of KwaZulu-Natal, Scottsville, Pietermaritzburg, South Africa; Oklahoma State University, UNITED STATES

## Abstract

Criniviruses accumulate in the phloem tissue and damage crops by reducing chlorophyll which is essential for plant growth and development. *Tomato chlorosis crinivirus* (ToCV) is vectored by several whitefly species that damage tomato crops throughout the world. In South Africa, ToCV is a poorly studied pathogen of global economic importance. Therefore, a national survey was initiated to investigate the occurrence and distribution of criniviruses infecting tomato crops in South Africa. Whitefly infested tomato crops exhibiting interveinal leaf chlorosis and chlorotic flecking symptoms were assayed for crinivirus infections using a multiplex reverse transcription polymerase reaction (RT-PCR) approach to assess for the presence of crinivirus species that are known to infect solanaceous hosts. Next-generation sequencing (NGS) was used to generate the complete genome of ToCV from South Africa. Results from the survey indicated that ToCV is presently the only crinivirus species infecting tomatoes in South Africa. Blast analysis showed that the RNA-1 segment of ToCV from South Africa (ToCR1-186) matched 99% to Spanish isolates. On the other hand, the RNA-2 (ToCR2-186) segment matched 98% to a South Korean isolate and three Spanish isolates. Although recombination events were not detected, phylogenetic studies showed inconsistencies in the grouping of RNA-1 and RNA-2 segments for some of the ToCV isolates analyzed in this study. Therefore, we suggest the possibility of intraspecific reassortment. This is the first comprehensive study and full genome sequence of ToCV from South Africa. The information generated from this study is intended to raise awareness of ToCV infections on tomato crops in South Africa.

## Introduction

Members of the genus *Crinivirus* belong to the family *Closteroviridae* and typically comprise a single-stranded bi-partite/tri-partite positive-sense RNA genome, approximately 15.6–17.9 kb in size [1]. The non-enveloped filamentous particles usually have two modal lengths (650–850 and 700–900 nm) and a diameter of 10–13 nm [1]. Criniviruses are exclusively transmitted by whiteflies belonging to the genera *Bemisia* and *Trialeurodes* in a semi-persistent manner [2]. More than 50 years ago Duffus [3], first reported a crinivirus infecting greenhouse cultivated cucurbits in California, since then, their emergence in field and greenhouse vegetable crop production has negatively impacted multi-million-dollar agricultural industries throughout the world [2; 4].

According to the World Tomato Processing Council (WPTC), tomatoes are the world’s largest vegetable crop category with an estimated 160 million tons produced in 2018 [5]. A short communication by Albuquerque et al. [6] highlights the threat and emergence of the crinivirus *Tomato chlorosis virus* (ToCV) on global tomato production. According to Simone et al. [7], reports of the ‘yellow leaf disorder’ as the disease resulting from ToCV infection dates back to 1989 from the Columbia and Suwannee counties in Florida (USA). Almost thirty years later, a viral disease that was incorrectly identified as a physiological and nutritional disorder has ‘silently plagued’ tomato fields and greenhouses throughout the world.

The European and Mediterranean Plant Protection Organization (EPPO) listed ToCV as a quarantined pathogen in some parts of the world [8]. Furthermore, they provided detailed information outlining the spread and severity of the disease in various countries and continents in which South Africa was mentioned although no official studies have been conducted to date. According to the Department of Agriculture, Forestry and Fisheries in South Africa (http://www.nda.agric.za), tomatoes are the second largest vegetable commodity after potatoes and constitute a staggering 24% of South Africa’s total vegetable production. Approximately 6000 hectares of land are used to grow tomatoes in South Africa during the summer months but production is limited to frost-free areas and greenhouses during winter.

In recent years, South Africa has experienced increasingly high whitefly populations that overwhelmed much of the country’s commercial and smallholder tomato farming communities [9]. *Bemisia* sp. and *Trialeurodes* sp. were primarily identified from basic phenotypic screening [9], however, further molecular assays are required to elucidate the species. Symptoms resembling those described in previous studies on the epidemiology and etiology of ToCV were observed in many of South Africa's major tomato growing areas [3; 7; 10; 11]. Symptomatic tomato plants typically exhibited interveinal leaf chlorosis, chlorotic flecking and leaf bronzing. Most of these symptoms developed in the mid to lower parts of tomato crops, and gradually progressed toward the apex as the plant matured. In addition, the onset of early senescence was observed. Although the fruit remained symptomless and is of marketable standards, many commercial farmers in South Africa reported a significant reduction in their overall yield.

Methods to suppress vector populations are a key management strategy for ToCV control as outlined by Tzanetakis et al. [2]. Various agro-ecological areas and microclimates across the globe possess a set of unique environmental parameters that directly or indirectly affect vector population dynamics. Apart from anthropogenic activity and farming practices that directly influence the density and diversity of insect pest populations, climate change variability is an integral element that many farmers and scientists have only recently recognized. In addition, it is almost impossible to control the spread of a virus without a profound understanding of its molecular genetics and factors that influence its virulence and distribution.

In Africa, ToCV was identified in Sudan, Morocco, and Tunisia located in the northern and Mediterranean parts of the continent. However, very limited information on ToCV is available from African countries [12; 13]. Recently, Moodley et al. [14] reported the occurrence of ToCV in South Africa. Against this background, a follow-up study was carried out to investigate the prevalence and epidemiology of criniviruses infecting tomato crops in South Africa. Additionally, a comprehensive analysis of the ToCV genome is described. It is hoped that the information generated from this study can be used to develop effective strategies to reduce the spread of ToCV.

## Materials and methods

### Sample collection

**Fig 1:**
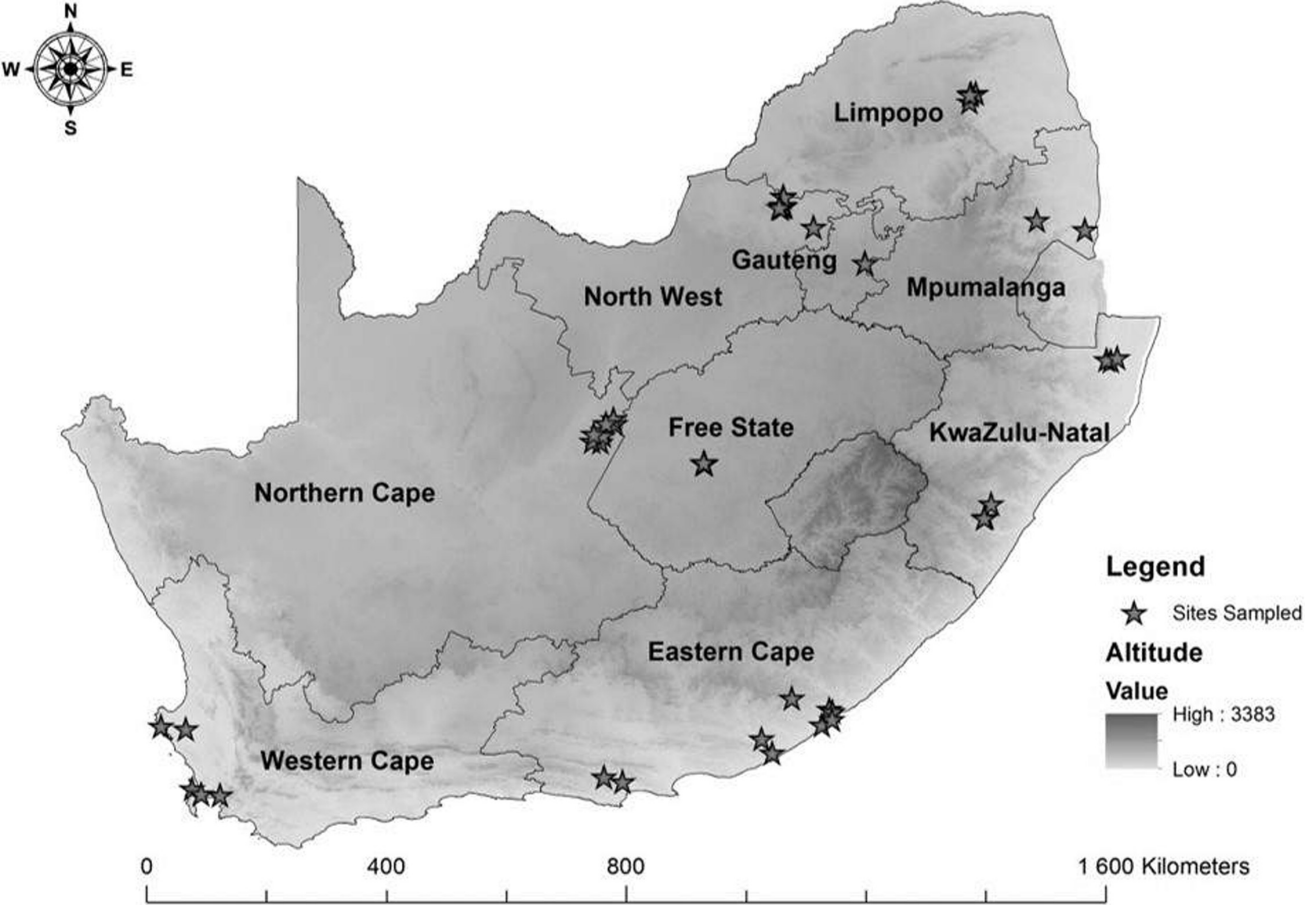
A map of the tomato farms surveyed in South Africa that was generated using GPS coordinates and ArcGIS v9.3 software. The exact GPS coordinates of each sampling site are represented by a star. Altitude is represented by varying intensities of grey that ranges from 0 meters above sea level (least intense shade of grey) to 3383 meters above sea level (most intense shade of grey).

Open field and greenhouse cultivated tomato crops were surveyed during the 2015 and 2016 growing seasons. Although tomato cultivation is concentrated in the Limpopo, Mpumalanga (Lowveld and Highveld), Northern KwaZulu Natal, Eastern Cape, and Western Cape provinces, all nine provinces were surveyed which included commercial, smallholder, and subsistence farms (Fig 1 and Table 1). A total of 787 tomato samples, and 182 weed samples exhibiting virus-like symptoms in the presence of whitefly populations, as well as asymptomatic leaf tissue, were collected and stored in zip-lock plastic bags. Only weed species occurring within proximity to field and greenhouse cultivated tomato crops were collected and analyzed. Each sample was appropriately labeled and assigned the corresponding GPS coordinates. A site map (Fig 1) of the survey was generated using ArcGIS v9.3 mapping software from the respective GPS coordinates listed in Table 1. Samples were placed on dry ice and transported to the University of KwaZulu Natal virology lab for further analysis.

**Table 1:** GPS coordinates of the tomato farms that were surveyed for ToCV infections.

Name (Farm)	Latitude	Longitude	Province
Duiwelskloof	-23,63961	30,07786	Limpopo
Duiwelskloof	-23,50725	30,17657	Limpopo
Mooketsie	-23,51224	30,10221	Limpopo
Rita ZZ2	-23,52945	30,08609	Limpopo
Johan Mare	-25,55161	31,81015	Mpumalanga
Visage	-25,41018	31,09415	Mpumalanga
Qoloko	-27,51811	32,20542	KwaZulu-Natal
Farm Z	-27,50542	32,13524	KwaZulu-Natal
Skoonsisters Bos	-33,04054	18,31801	Western Cape
Shelligerhout	-33,83642	24,8746	Eastern Cape
Rooinek	-33,76295	24,59784	Eastern Cape
Saldana Bay	-32,99778	17,94556	Western Cape
Pikoli	-32,98333	27,86667	Eastern Cape
Oranjezicht City Farm	-33,9422	18,4148	Western Cape
Philliphi	-34,01667	18,55	Western Cape
Helderberg farm	-34,03258	18,83369	Western Cape
Small Holder farm	-33,40305	27,12101	Eastern Cape
Stutterheim	-32,57874	27,4149754	Eastern Cape
Richmond	-29,86669	30,32378	KwaZulu-Natal
Jozini	-27,47556	32,29917	KwaZulu-Natal
Farm 1 (EC)	-32,74798	27,97904	Eastern Cape
Melody Farm	-32,78109	28,02095	Eastern Cape
Farm 2 (Tomato farm)	-33,18922	26,96126	Eastern Cape
Red Baron Tomatoes	-32,90275	28,01745	Eastern Cape
Ukulinga	-29,66415	30,40549	KwaZulu-Natal
Rive Lea	-29,88241	30,29718	KwaZulu-Natal
Delmot	-26,05597	28,50958	Gauteng
Mogwase	-25,22291	27,23971	North West
Mogwase	-25,19614	27,28241	North West
Mogwase	-25,05229	27,28241	North West
Brits	-25,51545	27,73865	Gauteng
Barkley West	-28,56164	24,58220	Northern Cape
Bloemfontein	-29,05453	26,10050	Free State

### Electron microscopy

A sterile scalpel was used to make an incision along the midrib of a fresh symptomatic leaf tissue sample. A leaf dip was performed by gently squeezing the exudate onto a droplet of sterile water. A copper formvar-coated grid was subsequently inverted onto the droplet for 5 min and negatively stained with a 3% potassium phosphotungstate solution for 1 min in the absence of light. Following a 20 min drying step, each copper grid was assessed for the presence of virus particles using a JOEL JEM-1400 transmission electron microscope.

### RNA extraction

RNA was extracted from 20 mg of frozen leaf tissue samples using a Quick-RNA MiniPrep kit (Zymo Research, USA) according to the manufacturers’ guidelines. Each sample was lysed in a sterile 1.5 ml microcentrifuge tube containing plastic beads, and 500 μl of lysis buffer, using a BioSpec Mini-Beadbeater-16 high-energy cell disrupter (USA). The column was subjected to a dry spin in the final step preceding elution, to remove unwanted residual ethanol from the wash buffer. A final elution of ≤ 30 μl was obtained and quantified using a nanodrop 1000 spectrophotometer (Thermo Fisher Scientific, USA).

### Virus detection using multiplex reverse transcription-polymerase chain reaction

First strand cDNA was synthesized from 500 ng of total RNA using a RevertAid Premium Reverse Transcriptase kit (Thermo Fisher Scientific Inc. USA). RNA templates were incubated at 65°C for 5 min and placed on ice prior to the addition of a master mix component as indicated by the manufacturer. A gene-specific Solanaceae reverse primer designed by Wintermantel and Hladky [15] was used at a final concentration of 1 ng/μl in each 20 μl RT reaction. Conditions for RT were 42°C for 1 h and 85°C for 10 min (enzyme inactivation).

*Tomato infectious chlorosis virus* (TICV), *Beet pseudo-yellows virus* (BPYV) and *Potato yellow vein virus* (PYVV) may produce similar or indistinguishable symptoms on tomato crops. To determine the possibility of infection by all known criniviruses, a multiplex system targeting the RNA-dependent RNA polymerase (RdRP) encoded by the ORF-1b gene was used [15]. PCR was performed in 20 μl volumes using a KAPA2G Fast HotStart ReadyMix PCR kit (KAPA Biosystems, USA) containing 0.75 ng/μl of each primer (Table 2), 30 ng of cDNA and 10 ul of KAPA master mix. Conditions for PCR were 95°C for 2 min followed by 35 cycles of 95°C for 25 s; 52°C for 30 s and 72°C for 25 s following a final elongation of 72°C for 10 min. PCR products were resolved on a 1.5% agarose gel containing SYBR Safe DNA Gel Stain (Invitrogen, USA).

**Table 2:** A set of multiplex RT-PCR primers designed by Wintermantel and Hladky [15], to distinguish criniviruses that infect solanaceous hosts.

Primer	Sequence 5ʹ-3ʹ	Expected band size
Solanaceae reverse primer	TGTTBGAYAACCAWGTGTT	-
BPYV forward primer	TGATGTCTGGTTTGATGACGGG	643 bp
TICV forward primer	AAGAATGGACCTACCCAG	995 bp
ToCV forward primer	GCACCCTGATTGGTTCTAAAC	265 bp
PYVV forward primer	ATCGTTCGTTCTCAACCG	514 bp

### Cloning and sequencing

PCR positive samples were validated by cloning (using a TA cloning kit [Invitrogen, USA] according to the manufacturers’ guidelines) and sequencing. Five positive amplicons were excised from the agarose gel, purified using a Zymoclean Gel DNA Recovery Kit (Zymo Research, USA) and ligated onto a PCR 2.1 cloning vector. The ligation reaction was incubated at 14°C overnight and kept on ice. Two microliters of each ligation reaction were added to TOP10 chemically competent *E*. *coli* cells and heat shocked at 42°C for 30 s. Successful transformants were selected (from blue/white colony screening) and cultured using LB (Luria-Bertani) broth in a shaking incubator at 37°C overnight. The overnight cultures were concentrated by centrifugation, and the recombinant plasmids were purified using a Zyppy Plasmid Miniprep Kit (Zymo Research, USA). The insert was confirmed using *Eco*R1 restriction enzyme digestion. All samples were sequenced in the forward and reverse direction at Inqaba Biotec (Pretoria, South Africa) using a 3500xL Genetic Analyzer (Applied Biosystems, USA). Sequences were analyzed using the BLAST tool in MEGA 6 software [16].

### Whole genome assembly and sequence analysis

Total RNA (≥ 50 ng/μl) was extracted from a ToCV positive sample using a Quick-RNA MiniPrep kit (Zymo Research, USA) and sequenced using an Illumina HiSeq 2500 Ultra-High-Throughput Sequencing System (Illumina Inc. USA) at the Agricultural Research Council Biotechnology Platform [ARC-BTP (Pretoria, South Africa)]. A paired-end library was generated using the platforms’ sequencing by synthesis technology (SBS). FastQC [17] was used to determine the read quality of NGS data generated from the HiSeq platform. The raw data was trimmed using Trimmomatics version 0.36, (parameters: Leading:14, Trailing:14, sliding window:4:15 minlen:70), where the low-quality sequence regions and Illumina universal adapter sequences were trimmed and removed. To remove the host sequence reads, the trimmed data were mapped against the reference genome: *Solanum lycopersicum* strain: Heinz 1706 (accession: GCF_000188115), where the whole genome sequence of *Solanum lycopersicum* strain: Heinz 1706 was downloaded from NCBI. Using the reference genome assembler: Bowtie2 version2.3.4 [18], an index reference database was constructed, and the sequence reads that did not align to the reference genome was collected for meta-assembly. The meta-assembly was done using the metaSPAdes pipeline within the de novo assembler: SPAdes version 3.11.1 [19]. Contigs were identified using Blast against the NCBI nucleotide database. ORF Finder (https://www.ncbi.nlm.nih.gov/orffinder/) was used to identify ORFs and translate proteins. Nucleotide and amino acid similarities of coding and non-coding regions were generated using the sequence identity and similarity tool SIAS (http://imed.med.ucm.es/Tools/sias.html). Phylogenetic analysis was conducted using MEGA 6 software. A best-fit model was generated from multiple sequence alignments for each RNA segment of the ToCV bipartite genome.

## Results

### Symptomatology

**Fig 2:**
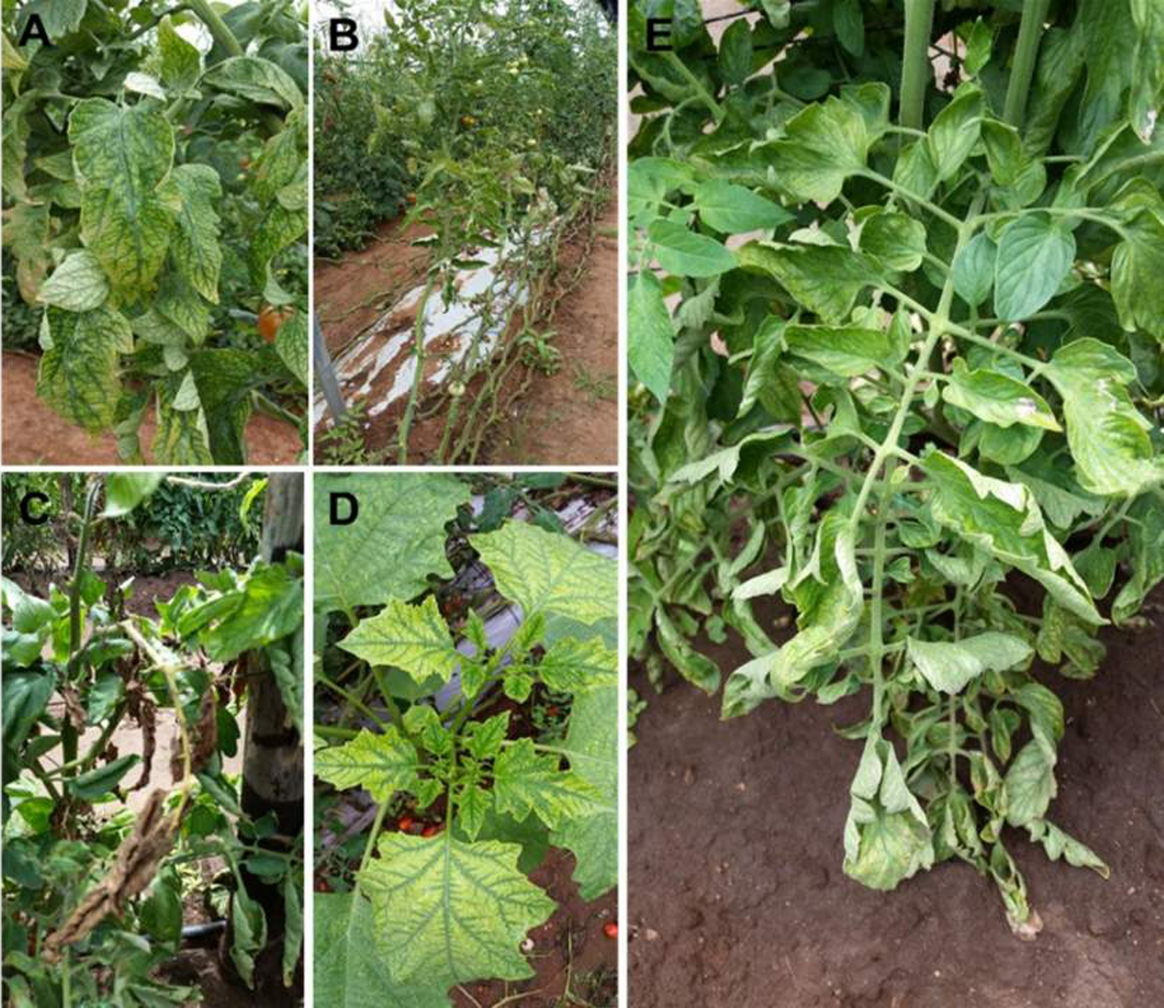
Symptoms typically associated with crinivirus infection. A: Interveinal leaf chlorosis, chlorotic flecking, and leaf bronzing symptoms on the lower mature leaves of tomato crops. B: Significant reduction in the yield and fruit size in symptomatic crops, however, fruit remain symptomless. C: Early senescence. D: The occurrence of weeds such as *Datura stramonium* growing along rows of tomato crops exhibiting interveinal leaf chlorosis and stunting in the presence of whiteflies. E: Reduced plant vigor, stunted growth, leaf brittleness, and upward leaf curling observed on lower mature leaves of whitefly-infested tomato crops.

Symptoms including interveinal leaf chlorosis, chlorotic flecking, and leaf bronzing that are typically associated with crinivirus infections were observed on tomato crops growing in fields and greenhouses located on commercial, smallholder, or subsistence farms (Fig 2). Although very widely distributed, these symptoms were concentrated in the northern, northwestern and central parts of South Africa where whitefly infestations of tomato crops were observed to be significantly higher. Whiteflies collected from field and tunnel sites across South Africa indicated the presence of *Bemisia* and *Trialeurodes* species from nymph and adult phenotypic analysis. Both whitefly genera are capable of transmitting criniviruses [20].

In addition to screening for crinivirus infection, a visual inspection of other known whitefly-transmitted viruses including torradoviruses, ipomoviruses, carlaviruses, and begomoviruses was carried out. Field observations showed that interveinal leaf chlorosis, chlorotic flecking, and leaf bronzing symptoms typically associated with crinivirus infection of tomatoes [10; 21] occurred more frequently amongst growers in South Africa [9] (Fig 2).

### Electron microscopy

**Fig 3:**
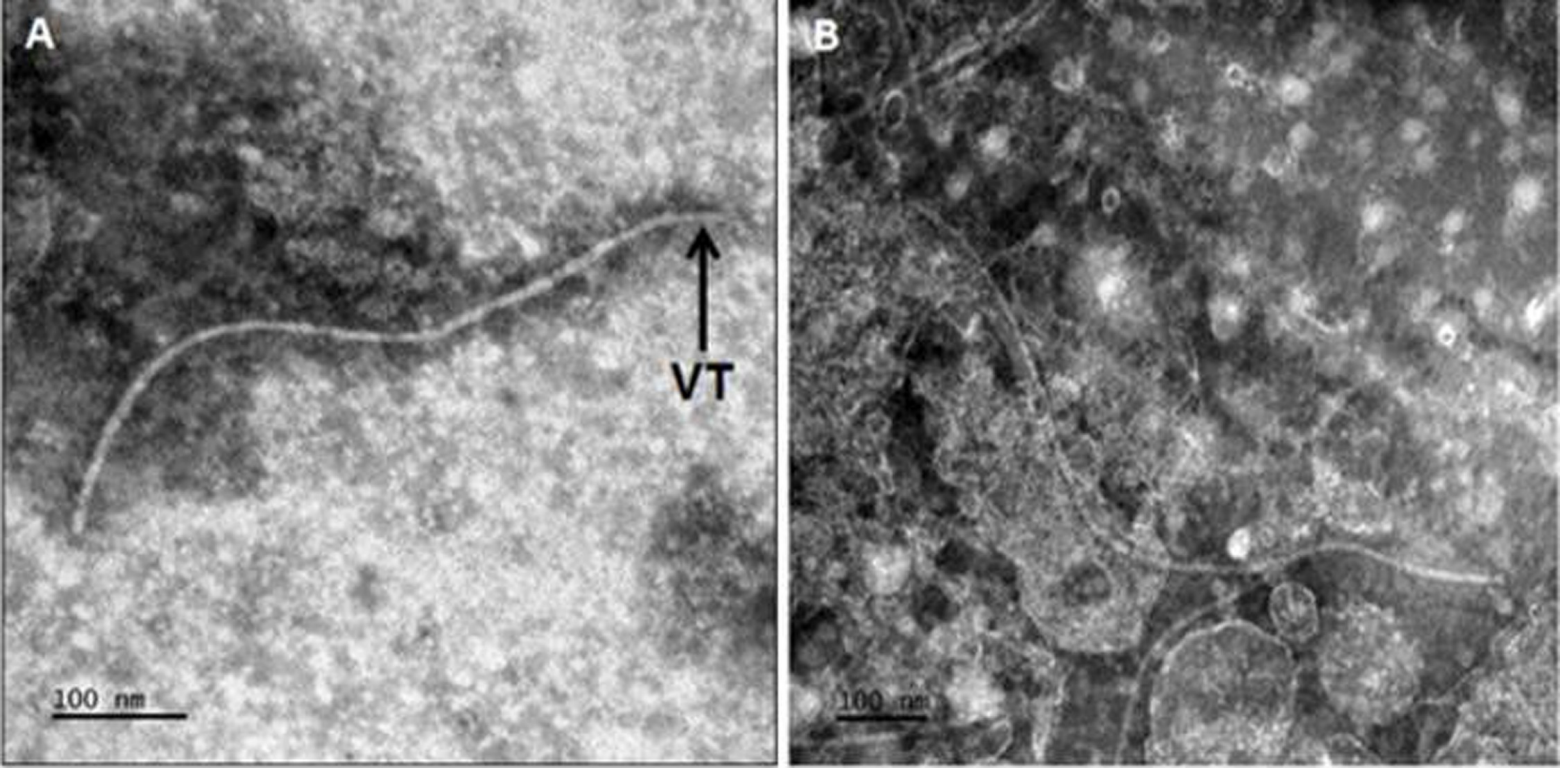
Electron micrograph showing long flexuous particles approximately 800 nm in length and 11 nm wide. A: An arrowhead structure resembling a virion tale (VT). B: Typical crinivirus ‘hair-like’ particles submerged in cellular debris from a crude tomato leaf extract.

Transmission electron microscopy (TEM) showed filamentous ‘hair-like’ virus particles approximately 800–900 nm in length and 10–12 nm wide (Fig 3), typically associated with members of the Closteroviridae family [1; 22].

### Virus detection using multiplex RT-PCR

Although multiplex RT-PCR assays were carried out to determine the presence of four crinivirus species (i.e. ToCV, TICV, BPYV, and PYVV) that are known to infect solanaceous hosts (Table 2) [15], our findings showed that only ToCV was detected in both field and greenhouse cultivated tomato crops and weeds (Table 3). In addition, *Solanum nigrum* (a short-lived perennial shrub occurring throughout South Africa) and *Datura stramonium* featured in Fig 2D (a category 1b invasive annual shrub species occurring throughout South Africa) tested positive for ToCV infection [14]. Sequence analysis of the 265 bp region located on the ORF-1b gene revealed a high level of similarity among all six ToCV isolates analyzed in this study with Nel-186Cr (KT989862) previously reported on *D*. *stramonium* in South Africa [14]. Blast analysis of the consensus sequence matched 98% to ToCV isolates from Sudan (JN411686) and Spain (KJ175084). Additionally, bell pepper (*Capsicum annuum* L.) crops were also screened for crinivirus infection, especially those in adjacent tomato farms, however, criniviruses were not detected on any of the samples from South Africa. In addition, NGS analysis of a pooled sample of RNA extractions from various symptomatic bell pepper crops infested with whiteflies confirmed the absence of any whitefly-transmitted viruses, suggesting that pepper-infecting strains of ToCV may not be present in South Africa.

**Table 3:** Prevalence of criniviruses infecting tomato crops (T) and weeds (W) in South Africa.

Area	Samples (T)	Samples (W)	ToCV (+)		TICV (+)		BPYV (+)		PYVV (+)	
			(T)	(W)	(T)	(W)	(T)	(W)	(T)	(W)
Limpopo	83	24	68 (81.9%)	12 (79.2%)	0	0	0	0	0	0
Eastern Cape	155	38	59 (38,1%)	8 (31,6%)	0	0	0	0	0	0
Mpumalanga	55	18	19 (34,5%)	5 (27,8%)	0	0	0	0	0	0
Western Cape	38	8	0	0	0	0	0	0	0	0
KwaZulu Natal	147	29	0	0	0	0	0	0	0	0
North West	132	15	87 (65,9%)	0	0	0	0	0	0	0
Gauteng	97	33	74 (76,3%)	13 (39.4%)	0	0	0	0	0	0
Northern Cape	0	0	0	0	0	0	0	0	0	0
Free State	80	17	64 (80%)	0	0	0	0	0	0	0
Total	787	182	371 (47,1%)	38 (21%)	0	0	0	0	0	0

^(+)–Virus-positive sample^

### Virus prevalence

Nucleic acid extracts from a total of 787 tomato samples and 182 weed samples from 15 species belonging to six botanical families (Amaranthaceae, Asteraceae, Brassicaceae, Euphorbiaceae, Malvaceae, and Solanaceae) were tested for crinivirus infection using multiplex RT-PCR to detect single or mixed infections of crinivirus species (ToCV, TICV, BPYV, and PYVV) that are known to infect solanaceous hosts. ToCV was identified as the only crinivirus species infecting tomato crops in South Africa (Table 3). In addition, ToCV was identified on two solanaceous weed species growing alongside tomato crops (Table 3).

Almost half (47%) of the tomato samples analyzed in this study tested positive for ToCV infections and the majority of these were collected from symptomatic tomato plants. Only two (*D*. *stramonium* and *S*. *nigrum*) of the 15 species of weeds collected in this study tested positive for ToCV infection which accounted for approximately 21% (38/182) of the samples analyzed. Both weed species belong to the Solanaceae family and can be found throughout much of South Africa. *D*. *stramonium* was the major weed species harboring ToCV in the northern (Limpopo, Gauteng) and the southeastern (Eastern Cape) parts of the country. To a lesser extent, *S*. *nigrum*, growing among tomato crops in the north-eastern parts of the Mpumalanga province tested positive for ToCV infection. Both ToCV positive weed species *D*. *stramonium* and *S*. *nigrum* were symptomatic. ToCV was not identified from any of the asymptomatic weed samples that were infested with whiteflies.

### Genome analysis

**Fig 4:**
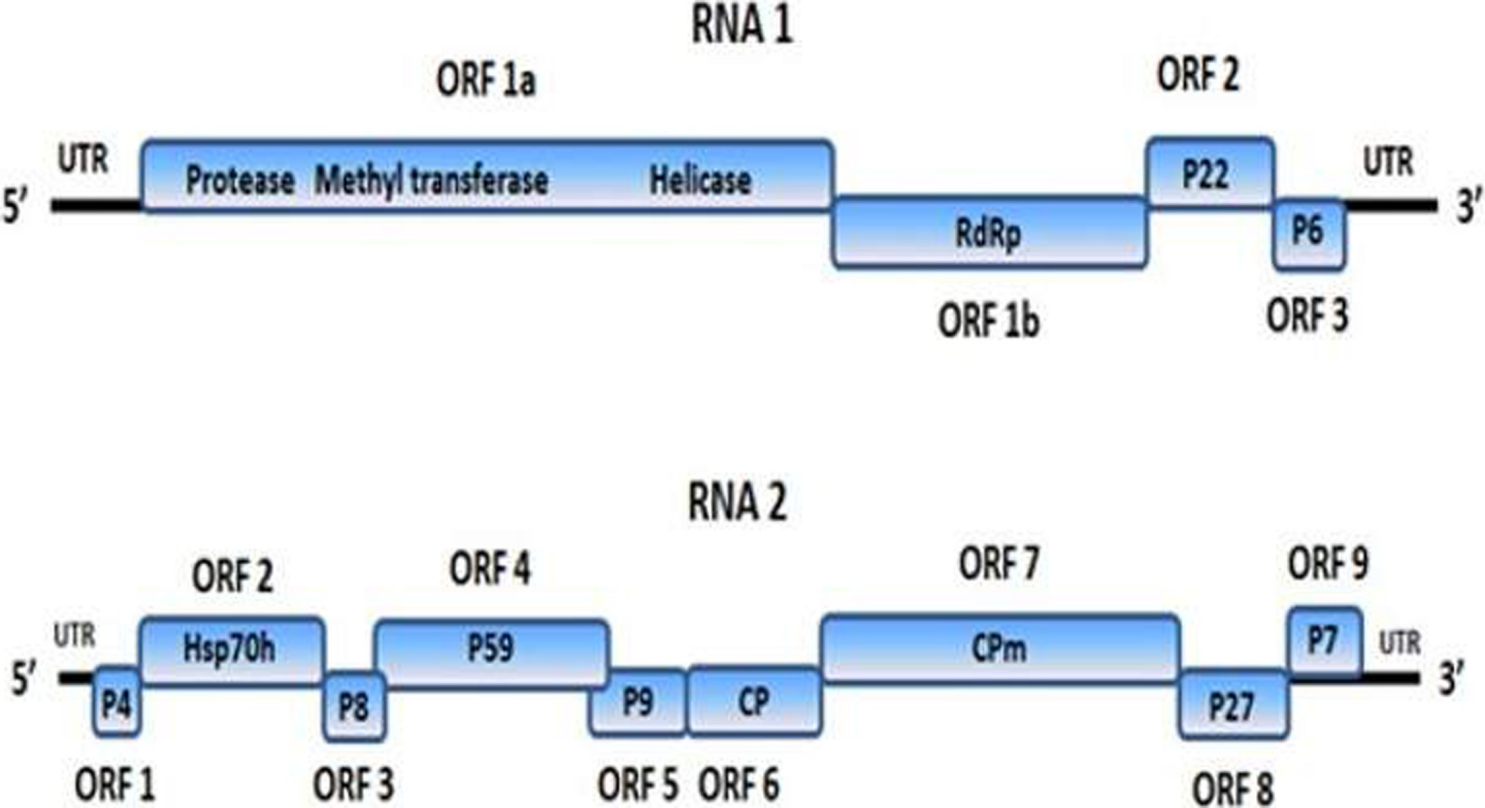
Bi-partite genome organization of ToCV. **The relative size and position of each ORF are represented accordingly.** Four ORFs on the RNA-1 segment encodes replication proteins, a suppressor of gene silencing (P22) and a putative protein (P6) with a transmembrane domain. RNA-2 encodes a Heat shock protein homolog 70 (Hsp70h), Coat protein (CP) and a Minor coat protein (CPm) in addition to six putative proteins (P4, P8, P9, P27, and P7).

The use of high-throughput sequencing was a rapid and effective method to generate the full genome sequence of ToCV from South Africa. A total of 127,654 contigs were generated from the de novo metaSPAdes assembly. Only 2,101 contigs were not aligned to the *Solanum* genomes. Of the 2,101 contigs, 68 contigs were similar to known viral sequences. Contigs that matched to ToCV RNA-1 and RNA-2 sequences as per NCBI GenBank, ranged from 98% to 99%. RNA-1 spanned a total of 8610 nt in length comprising four ORFs, a 303 nt untranslated leader sequence at the 5ʹ end, and a 190 nt non-coding region at the 3ʹend (Fig 4). ORF-1a (5838 nt/1945 aa) located at position 304 to 6141 encodes a 221 kDa multifunctional protein comprising a protease, methyltransferase and helicase domain. The 59 kDa RNA-dependent RNA polymerase (RdRp) encoded by ORF-1b (nucleotides 6212–7657) is likely expressed as a result of a +1 ribosomal frameshift at the end of ORF-1a; a common strategy among members of the Closteroviridae family [23]. In addition, all eight motifs, consistent with findings of Koonin [24] were identified. ORFs 1a and 1b translate proteins involved in virus replication. ORF-2 (7664–8245) encodes a 22 kDa protein (P22) which is a suppressor of gene silencing [25], and ORF-3 (8265–8420) encodes a 6 kDa protein (P6) at the 3ʹ terminus of RNA-1, possessing a transmembrane domain similarly recognized in other crinivirus species [23]. Blast analysis of the RNA-1 sequence ToCR1-186 (GenBank accession no. KY471129) from South Africa showed the highest (97–98%) sequence identity to Spanish isolates AT80/99-IC (KJ740256), AT80/99 (DQ983480) and PI-1-2 (KJ200308). Table 4 shows a detailed comparison of the nucleotide and amino acid similarity profiles of coding and non-coding regions of ToCR1-186 and the fully sequenced RNA-1 segment of nineteen ToCV isolates that have been reported from various locations around the world. Although all values among all isolates were greater than 90% much of the individual ORFs and UTRs of ToCR1-186 shared the highest similarity to the Spanish isolates mentioned above.

**Table 4:** Nucleotide (nt) and amino acid (aa) similarities expressed as a percentage (%) for four ORFs and the none-coding regions on the ToCV RNA-1 segment.

ToCV Isolate	5ʹ UTR	ORF 1a		ORF 1b RdRp		ORF 2 P22		ORF 3 P6		3ʹNTR
	nt (%)	nt (%)	aa (%)	nt (%)	aa (%)	nt (%)	aa (%)	nt (%)	aa (%)	nt (%)
ToCR1-186 (KY471129)	100	100	100	100	100	100	100	100	100	100
AT80/99-IC (KJ740256)	97.7	96.04	97	99.37	98.54	97.59	95.85	98.71	96.07	92.63
AT80/99_(DQ983480)	97.7	96.09	97	99.3	98.33	97.59	95.85	98.71	96.07	92.63
Pl-1-2 (KJ200308)	96.72	95.73	96	99.3	98.33	97.42	94.3	97.43	94.11	92.1
MM8 (KJ200306)	96.72	95.57	96	99.17	98.12	97.42	94.81	98.07	94.11	92.1
HP (KP114530)	96.39	93.16	96	98.06	95.63	96.21	92.22	98.71	96.07	92.63
HS (KP137098)	96.06	92.9	96	97.85	95.21	96.04	92.22	98.71	96.07	92.63
JJ5 (KP114527)	96.06	92.95	96	97.85	95.01	95.7	91.19	98.71	96.07	92.63
SDSG_(KC709509)	96.72	92.85	96	97.92	95.21	96.04	91.7	98.71	96.07	92.63
JJ (KP137100)	95.4	92.69	96	97.92	95.21	95.53	92.22	98.71	96.07	92.63
IS29 (KP114538)	96.06	92.54	96	97.92	95.42	96.21	92.22	98.71	96.07	92.1
IS17_(KP114535)	96.06	92.54	96	97.92	95.42	96.21	92.22	98.71	96.07	92.1
YG (KP114526)	96.06	92.54	96	97.92	95.42	96.21	92.22	98.71	96.07	92.1
2.5 (KJ200304)	95.4	92.69	96	97.85	95.21	95.87	91.7	98.07	96.07	91.57
ToC-Br2_(JQ952600)	95.08	92.49	96	97.85	95.21	96.04	91.7	98.71	96.07	91.05
Gr-535 (EU284745)	96.06	92.49	96	97.85	95.21	95.53	90.67	98.71	96.07	92.63
ToCV-BJ (KC887998)	96.06	92.54	96	97.85	95.01	95.7	92.22	98.71	96.07	92.63
ToCV-RNA1 (AY903447)	96.06	92.59	96	97.85	95.21	95.7	91.7	98.71	96.07	92.63
NJ (KF018280)	96.39	90.43	94	97.99	95.63	95.87	91.19	98.07	94.11	92.63

The 8242 nt RNA-2 molecule comprised of a 237 nt 5ʹ leader sequence, nine ORFs and a 214 nt 3ʹ untranslated region (Fig 4). ORF-1 (nucleotides 238–339) encodes a 4 kDa protein that may function as a transmembrane protein because of the large hydrophobic domain [26]. The 61.9 kDa heat shock protein 70 homolog (Hsp70h) encoded by ORF-2 at nucleotides 732–2396 is a conserved protein among species of the genus *Crinivirus*, and functions in the cell-to-cell movement, virus assembly and may associate with virion tails. ORF-3 (nucleotides 2406–2609) encodes a putative 8 kDa protein with no known function to date. A 59 kDa protein (P59), thought to associate with virion tails and movement is encoded by ORF-4 located at nucleotide position 2561–4114. The putative 9 kDa (P9) protein encoded by ORF-5 (nucleotides 4096–4332) has no assigned function. A 29 kDa coat protein (CP) encoded by ORF-6 (nucleotides 4332–5105) encapsidates the major part of ToCV virions whilst the 76 kDa minor coat protein (CPm) encoded by ORF-7 (nucleotides 5111–7120) encapsidates the 5ʹ end and is associated with virion tails and possibly cell-to-cell movement [26]. Lozano et al. [27] relate to a study in which the CPm of *Lettuce infectious yellows virus* (LIYV) was linked to vector transmission [28]. ORF-8 (nucleotides 7124–7822) encodes a 27 kDa (P27) protein that is conserved among members of the Crinivirus genus, however, the amino acid similarity among species is low [27]. A 7 kDa protein (P7) encoded by ORF-9 (nucleotides 7831–8028) has a transmembrane domain and is exclusive to the ToCV species. The functional role of this protein is presently not known. Blast analysis of the RNA-2 segment ToCR2-186 (GenBank accession no. KY471130) from South Africa matched 98% with the isolate JJ (KP137101) from South Korea and the Spanish isolates AT80/99-IC (KJ740257), MM8 (KJ200307), 2.5 (KJ200305) and AT80/99 (DQ13614). A higher level of consistency was observed in the non-coding regions of RNA-2 (Table 5) than in RNA-1 (Table 4). In addition, nucleotide and amino acid similarity profiles among individual ORFs were higher in RNA-2 as compared to RNA-1. Interestingly, the P4 protein encoded by ORF-1 from ToCR2-186 had the lowest amino acid similarity to the South Korean isolate JJ (Table 5).

**Table 5:** Nucleotide (nt) and amino acid (aa) similarities expressed as a percentage for nine ORFs and the none-coding regions on the ToCV RNA-2 segment.

ToCV Isolate	5ʹUTR	ORF 1 P4	ORF 2 Hsp70h	ORF 3 P8	ORF 4 P59	ORF 5 P9	ORF 6 CP	ORF 7 CPm	ORF 8 P27	ORF 9 P7	3ʹUTR
	nt	nt (aa)	nt (aa)	nt (aa)	nt (aa)	nt (aa)	nt (aa)	nt (aa)	nt (aa)	nt (aa)	nt
ToCR2-186	100	100 (100)	100 (100)	100 (100)	100 (100)	100 (100)	100 (100)	100 (100)	100 (100)	100 (100)	100
JJ (KP137101)	97.89	96.07 (90.90)	98.97 (97.65)	97.54 (92.53)	98.06 (95.55)	99.15 (97.43)	98.96 (97.27)	97.61 (94.19)	97.99 (96.55)	97.47 (93.84)	98.63
AT80/99-IC (KJ740257)	97.47	99.01 (96.96)	98.61 (96.75)	97.05 (92.53)	97.94 (94.97)	98.73 (96.15)	98.57 (96.49)	97.31 (94.46)	98.28 (97.41)	97.97 (95.38)	97.71
MM8 (KJ200307)	97.89	99.01 (96.96)	98.55 (96.93)	97.05 (92.53)	98.13 (95.35)	99.15 (97.43)	98.57 (96.49)	97.16 (97.17)	98.28 (97.41)	97.97 (95.38)	97.71
2.5 (KJ200305)	97.47	99.01 (96.96)	98.67 (96.93)	97.54 (92.53)	97.87 (94.77)	99.15 (97.43)	98.44 (96.1)	97.21 (94.31)	98.14 (97.41)	97.97 (95.38)	97.71
AT80/99 (DQ136146)	97.89	99.01 (96.96)	98.67 (96.93)	97.54 (92.53)	97.81 (94.77)	99.15 (97.43)	98.44 (96.1)	97.31 (94.46)	98.28 (97.41)	97.97 (95.38)	97.71
Pl-1-2 (KJ200309)	97.89	99.01 (96.96)	98.43 (96.2)	97.05 (91.04)	97.61 (94.19)	99.15 (97.43)	98.83 (96.88)	97.06 (94.31)	98.14 (96.98)	97.47 (93.84)	97.71
YG (KP114536)	97.47	99.01 (96.96)	98.37 (95.3)	96.56 (89.55)	98 (94.97)	99.15 (97.43)	98.32 (96.1)	96.66 (92.37)	98.28 (95.68)	97.97 (96.92)	99.08
HP (KP114537)	97.47	99.01 (96.96)	98.43 (95.48)	96.56 (89.55)	98 (94.97)	99.15 (97.43)	98.32 (96.1)	96.61 (92.37)	97.99 (95.25)	97.97 (96.92)	99.08
JJ5 (KP114534)	97.47	99.01 (96.96)	98.37 (95.3)	96.56 (89.55)	98 (94.97)	99.15 (97.43)	98.32 (96.1)	96.66 (92.37)	98.28 (95.68)	96.96 (93.84)	97.71
JJ3 (KP114533)	97.47	99.01 (96.96)	98.37 (95.3)	96.56 (89.55)	98 (94.97)	99.15 (97.43)	98.32 (96.1)	96.61 (92.22)	98.28 (95.68)	96.96 (93.84)	97.71
SDSG (KC709510)	97.89	98.03 (93.93)	98.31 (95.3)	96.56 (91.04)	97.94 (95.16)	98.73 (96.15)	98.19 (95.71)	96.56 (92.07)	98.42 (96.12)	97.97 (96.92)	97.71
HS (KP137099)	97.05	99.01 (96.96)	98.37 (95.3)	96.56 (89.55)	97.94 (94.97)	99.15 (97.43)	98.32 (96.1)	96.61 (92.37)	98.28 (95.68)	96.96 (93.84)	97.71
IS29 (KP114529)	98.31	98.03 (93.93)	98.19 (95.3)	97.05 (91.04)	97.87 (94.39)	98.73 (96.15)	98.19 (95.71)	96.31 92.52)	97.71 (94.39)	97.97 (96.92)	99.08
IS17 (KP114525)	98.31	98.03 (93.93)	98.19 (95.3)	97.05 (91.04)	97.87 (94.39)	98.73 (96.15)	98.19 (95.71)	96.26 (92.37)	97.99 (94.82)	97.47 (95.38)	97.71
Gr-535 (EU284744)	97.47	97.05 (93.93)	97.83 (94.4)	97.05 (91.04)	97.94 (94.77)	99.15 (97.43)	97.93 (95.33)	96.36 (92.07)	97.99 (94.82)	97.97 (96.92)	98.17
ToCV RNA2 (AY903448.1)	97.89	97.05 (90.9)	98.13 (95.12)	97.05 (91.04)	97.55 (93.81)	98.73 (96.15)	98.19 (95.71)	96.31 (91.47)	98.42 (95.68)	97.97 (96.92)	94.97
ToCV RNA2 (KJ815045)	97.89	98.03 (93.93)	98.01 (94.76)	96.07 (91.04)	98 (94.77)	98.73 (96.15)	98.19 (95.71)	96.36 (91.62)	98.28 (95.25)	97.97 (95.38)	94.06
ToCV-BJ (KC887999)	97.89	98.03 (93.93)	98.13 (95.3)	96.56 (91.04)	97.36 (94.58)	97.89 (93.58)	98.32 (96.1)	96.36 (91.62)	98.28 (96.12)	97.47 (95.38)	97.71
ToC-Br2 (JQ952601)	98.31	98.03 (93.93)	97.95 (94.4)	96.07 (91.04)	97.81 (94.19)	99.15 (97.43)	97.8 (95.33)	96.11 (92.07)	97.99 (94.82)	97.97 (96.92)	98.63

### Phylogeny

**Fig 5:**
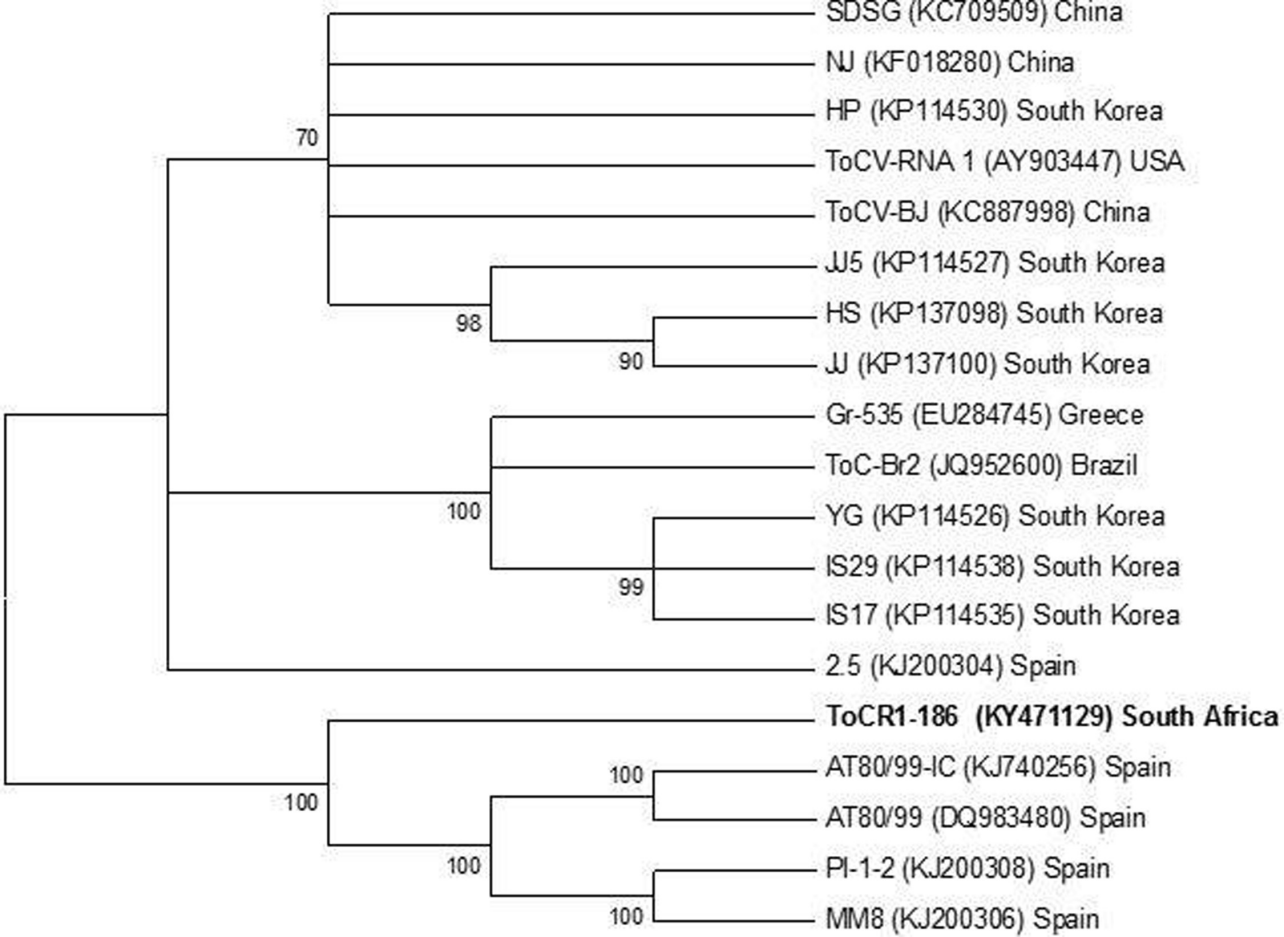
Phylogenetic relationship of the RNA-1 segment of Tomato chlorosis virus from South Africa (ToCR1-186) and all other fully sequenced ToCV isolates to date. The phylogram was constructed using the Maximum likelihood method based on the Tamura-Nei model and a bootstrap value of 1000 replicates in MEGA 6. The accession number for each isolate is displayed in parenthesis.

**Fig 6:**
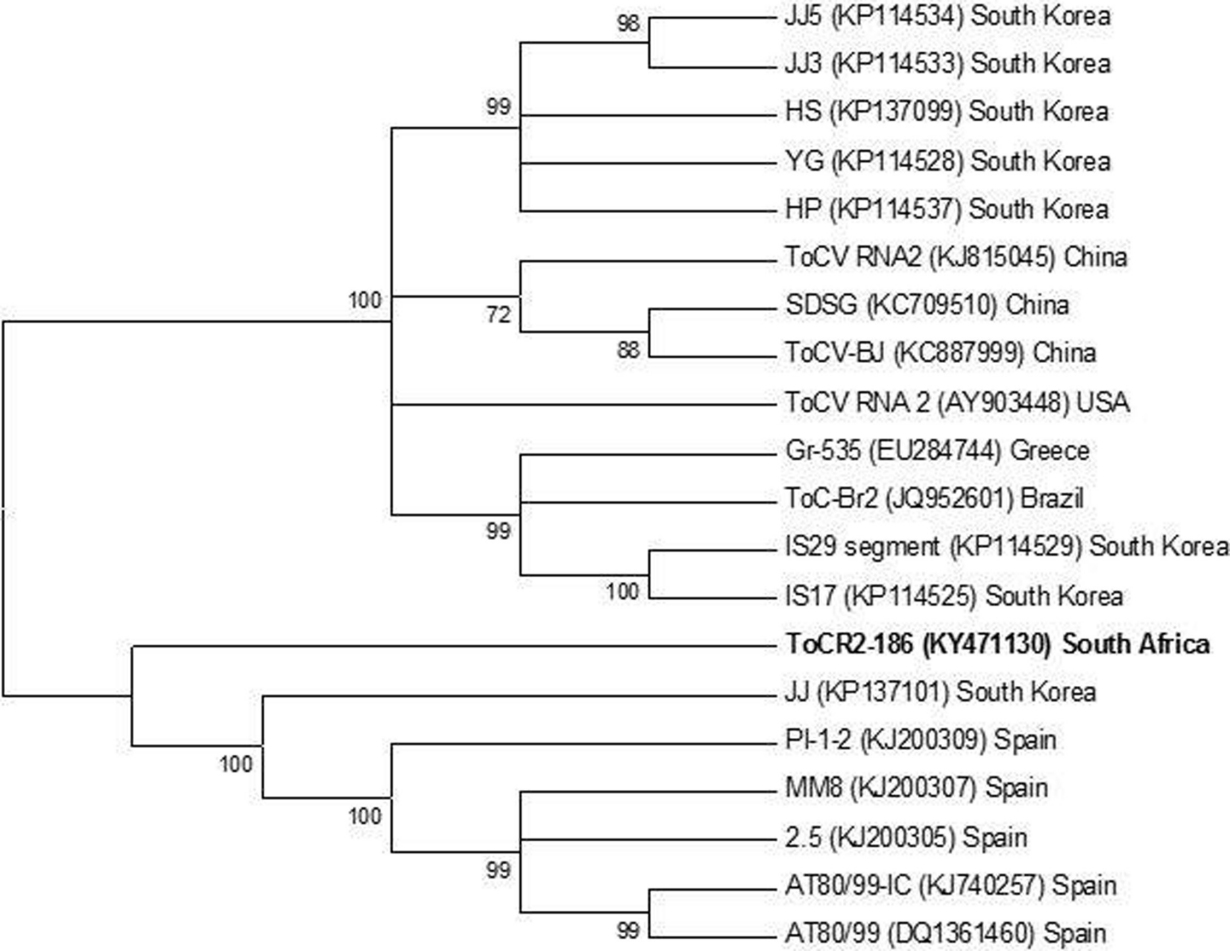
Phylogenetic relationship of the RNA-2 segment of Tomato chlorosis virus from South Africa (ToCR2-186) and all other fully sequenced ToCV isolates to date. The phylogram was constructed using the Maximum likelihood method based on the Hasegawa-Kishino model and a bootstrap value of 1000 replicates in MEGA 6. The accession number for each isolate is displayed in parenthesis.

The complete sequence of ToCV from South Africa was elucidated using next-generation sequencing (NGS). A consensus sequence was generated from the low nucleotide variability among ToCV isolates infecting weeds and tomato samples. Phylogenetic analysis of the RNA-1 segment showed that the South African ToCV isolate ToCR1-186 (Accession no. KY471129) was closely related to the group of Spanish isolates but did not cluster with any of these isolates (Fig 5). These relationships are supported by strong bootstrap values (100%). Interestingly, the RNA-2 segment from South Africa (ToCR2-186; Accession no. KY471130) diverged from the South Korean Isolate JJ (which grouped with Spanish isolates) but did not group with these isolates (Fig 6). The relationship between Spanish isolates and the Korean isolate JJ is supported by strong bootstrap values, however, their relationship with ToCR2-186 is not supported by a significant bootstrap value.

Similarly, ToCR2-186 did not cluster with any of the isolates and formed a unique clade. The RNA-1 segment of the ToCV isolate JJ groups exclusively with other South Korean isolates (HS, JJ5) and clusters with isolate HS. Inconsistencies in the grouping of the RNA-1 and RNA-2 segments belonging to the same ToCV isolate suggest the possibility of intraspecific genetic acquisition through reassortment and recombination. South Korean isolates displayed the highest level of diversity and seem to show this trend more frequently than others (Figs 5 and 6).

## Discussion

Wintermantel and Wisler [21] demonstrated that hosts infected with different crinivirus species may produce the same symptoms. For example, tomatoes infected with TICV or ToCV are indistinguishable based on visual inspection. These symptoms may also be mistaken for physiological disorders and pesticide toxicity. In some cases, it may simply be a nutrient deficiency or some abiotic stress. Therefore, it is important to use molecular methods to validate symptoms in the field. In addition, criniviruses that are capable of infecting solanaceous hosts have been grouped accordingly and include ToCV, TICV, BPYV, and PYVV [15]. For these reasons, a multiplex RT-PCR approach was used to detect single or mixed infections of crinivirus species that commonly infect solanaceous hosts. Our results showed that only *Tomato chlorosis crinivirus* (ToCV) was identified from leaf tissue samples collected during this survey (Table 3).

The isolate of ToCV previously reported from South Africa (Nel-186Cr; Accession no. KT989862) on *D*. *stramonium* [14], was predominant among tomato crops and some arable weed species. All subsequent aligned ToCV sequences analyzed in this study matched ≥ 99% to Nel-186Cr. The low nucleotide sequence variability among ToCV isolates infecting weeds and tomato crops in South Africa raises concerns about the epidemiology of the virus and suggests that ToCV isolates identified on some weed species are likely to be the same as those infecting nearby tomato crops. Although weed samples belonging to six botanical families were assayed for crinivirus infections, only two weed species i.e. *S*. *nigrum* and *D*. *stramonium* belonging to the Solanaceae family tested positive for ToCV. Importantly, these weeds are distributed throughout much of South Africa and may have significantly contributed to the epidemiology of ToCV.

According to Farina et al. [29], it is well known that the development of plant disease epidemics begins with viruliferous vectors that have acquired viruses from primary sources of inoculum such as weeds and subsequently spread to nearby crop fields. Further development of the epidemic is facilitated by a process known as secondary spread. Macedo et al. [30] demonstrated the importance of primary spread in the epidemiology of ToCV on tomato crops, and how the spatial dynamics of the disease can be contained via intervention. Therefore, understanding the role of primary spread is fundamental to the development of effective cultural methods that reduce sources of inoculum. Further studies are required to determine the transmission efficiency of ToCV from infected *S*. *nigrum* and *D*. *stramonium* hosts to cultivated tomato varieties in South Africa. This will provide further insight into the impact of these weed species as reservoirs of ToCV in South Africa’s tomato industry.

A high prevalence of ToCV was identified in the northern, north-western and central parts of South Africa, and to a lesser extent, in the north-east and southeastern regions (Table 3). Interestingly, ToCV was not identified in tomato growing regions along the central east coast (KwaZulu Natal Province), further west of the Eastern Cape province (Port Elizabeth) and in the Western Cape province, even though large whitefly populations and symptoms characteristic for crinivirus infections were observed suggesting the likelihood of nutrient deficiencies or physiological disorders. Moreover, only weed samples collected from the northern and south-eastern parts of the country tested positive for ToCV; with a higher prevalence in the north (Table 3). Although altitude did not significantly impact the prevalence of ToCV in South Africa, the warmer, drier conditions in the north seemed to favor the population dynamics of the whiteflies and the virulence of ToCV (Fig 1 and Table 3).

The small percentage of asymptomatic tomato samples that tested positive for ToCV infections may be the result of early or latent stages of infection. Since tomato samples were collected in the presence of whiteflies it was necessary to test all samples (symptomatic/asymptomatic) to reduce the disparity/bias associated with prevalence studies. Some tomato samples that tested positive for ToCV exhibited symptoms atypical to those caused by ToCV and was later found to be coinfected with an array of other viruses including *Tomato torrado virus* (ToTV), *Pepino mosaic virus* (PepMV), and *Potato virus Y* (PVY). These viruses were serendipitously identified using next-generation sequence analysis. Coinfecting viruses may have suppressed the rate of ToCV replication and subsequent symptom expression in a phenomenon known as virus synergism.

ToCV can be vectored by five types of whiteflies with varying levels of efficiency [2]. *Trialeurodes abutilonea* and *Bemisia tabaci* (MEAM1, formerly biotype B) are highly effective vectors in comparison to *Trialeurodes vaporariorum* and *Bemisia tabaci* (New World, formerly biotype A) [21]. Navas-Castillo et al. [31] reported the emergence and spread of ToCV by a highly effective *Bemisia tabaci* (MED, formerly biotype Q) in Europe. In this study, mixed whitefly populations belonging to the genera *Bemisia* and *Trialeurodes* were identified in open field and greenhouse-produced tomatoes that tested positive for ToCV infection. *Bemisia* sp. were more predominant throughout the areas surveyed; however, significantly higher populations of *Trialeurodes* sp. were observed in most greenhouses and sampling sites located in the North-West province.

A further investigation of the molecular characteristics of ToCV from South Africa provided insight into the possibility of genetic recombination and reassortment. Phylogenetic analysis (Figs 5 and 6) shows that the RNA-1 segment (ToCR1-186) groups most closely with Spanish isolates, however, the RNA-2 (ToCR2-186) diverges from and shows the highest identity with South Korean isolate JJ which ultimately group with Spanish isolates. Interestingly, the RNA-1 segment of the South Korean isolate JJ clusters within a group of South Korean isolates. Similar trends are observed with South Korean isolate HP (RNA-1 groups with Chinese isolates and RNA-2 groups with South Korean isolates) and Spanish isolate 2.5 (RNA-1 groups independently and RNA-2 clusters within the Spanish group). This also suggests a possible connection between Spanish and some South Korean isolates, specifically JJ. This genetic diversity of the RNA-1 and RNA-2 segments of ToCV isolates throughout the world may have resulted from recombination. However, no conclusive recombinant events were detected following the *in-silico* analysis of these RNA molecules using recombination detection program (RDP 4) [32]. Subsequently, our findings suggest the likelihood of intraspecific reassortment, based on the inconsistencies associated with the position of the RNA-1 and RNA-2 segments of ToCV isolates from phylogenetic analysis (Figs 5 and 6)

The grouping of the South African ToCV isolate with Spanish isolates (Figs 5 and 6) indicates that the disease may have spread from Spain into the Mediterranean and Sudan [9]. The establishment of the exotic Mediterranean whitefly biotypes Q (MED) and B (MEAM1) in South Africa may have displaced indigenous species by means of fitness and resistance to broad-spectrum pesticides that indirectly killed off natural enemies [33]. Apart from the movement of contaminated plant and seed material across countries and farming communities around the world, the spread of ToCV in South Africa is exacerbated by farming practices and pesticide abuse. Smallholder and rural farmers generally lacked resources and knowledge of disease management. Much of their efforts were further hampered by the absence of skilled government extension workers able to bridge the gap between the technical jargon associated with research and farmers.

Since epidemics and emerging disease are strongly influenced by the density and diversity of vector populations (especially with viral pathogens such as ToCV that are not mechanically or seed transmissible), a greater understanding of what influences vector dynamics is required. Tzanetakis et al. [2] indicate that the management of ToCV is primarily by chemical and cultural methods. Conversely, chemical control is considered somewhat inefficient since whitefly vectors can still transmit viruses before being poisoned with pesticides. Consequently, alternative hosts such as weeds cannot be overlooked as potential sources of virus inoculum. They serve as overwintering hosts that reduce the quality of soils in arable land. Their robust nature and adaptive capacity to survive and reproduce in adverse conditions is an evolutionary trend that indirectly contributes to the epidemiology of vectors such as whiteflies and viruses such as ToCV throughout the world. On the other hand, climate variability directly influences the level of biotic and abiotic stress in agricultural systems. Knowledge of abiotic factors that contribute to pest and disease outbreaks is fundamental to the development of climate models that project the risks associated with epidemics. Pest phenology models may be an effective way to reduce vector populations and pesticide applications when coupled with cultural, chemical, and biological practices. Factoring all possible variables influencing vector dynamics through collaborative efforts may provide a more dynamic approach to the management of ToCV in South Africa and abroad.

## Conclusions

ToCV is the most abundant and widespread whitefly-transmitted virus infecting tomato crops in South Africa and results in millions of dollars in crop losses throughout the world. Until genetic resistance is available, ToCV can be managed by integrating chemical and biological (particularly in greenhouses) control with cultural practices such as good crop husbandry, sticky traps, pheromones, and crop rotation.

Insecticide treatment to control vector populations must be used sparingly and rotated with different active ingredients (AI’s) to minimize treadmill effects. The removal of alternative sources of inoculum such as weeds, crop residues, and volunteer plants prior to planting is also recommended to reduce primary spread of ToCV. Parasitic wasps belonging to the species *Encarsia formosa* can be used to effectively control vector populations in greenhouse cultivated tomato crops. Optimizing the use of cultural and biological control measures will ultimately lead to a decrease in pesticide applications and may have positive outcomes on the long-term sustainability of whitefly population control and virus management.

Additionally, we recommend the use of climate/pest phenology models that can provide valuable information on the distribution and population dynamics of whitefly vectors. This early warning system serves as part of a decision-making process to initiate planting or implement control measures. More importantly, stringent policies on the trade and movement of plant material across borders, together with a strategic management approach can significantly reduce the economic impact of ToCV infections throughout the world.
